# Functional Recovery and Emotional Burden After Burn Injury: A Quality of Life Assessment in Romanian Burn Survivors

**DOI:** 10.3390/diseases14060212

**Published:** 2026-06-11

**Authors:** Andreea Ungureanu, Maria-Cristina Marinescu, Adriana-Nicoleta Trandafir, Valeria Coviltir, Carmen Giuglea, Silviu-Adrian Marinescu

**Affiliations:** 1 Doctoral School, Carol Davila University of Medicine and Pharmacy, 8 Eroii Sanitari Boulevard., 050474 Bucharest, Romania; 2 Department of Plastic and Reconstructive Surgery, Carol Davila University of Medicine and Pharmacy, 8 Eroii Sanitari Boulevard., 050474 Bucharest, Romania; 3 Discipline of Medical Physiology, Faculty of Medicine, Carol Davila University of Medicine and Pharmacy, 8 Eroii Sanitari Boulevard., 050474 Bucharest, Romania; 4 Department of Plastic and Reconstructive Surgery, ‘Carol Davila’ Central Military Emergency Hospital, 88 Mircea Vulcănescu Street, 10825 Bucharest, Romania; 5 Department of Ophthalmology, Faculty of Medicine, Carol Davila University of Medicine and Pharmacy, 8 Eroii Sanitari Boulevard., 050474 Bucharest, Romania; 6 Department of Plastic and Reconstructive Surgery, ‘Bagdasar-Arseni’ Emergency Hospital, 12 Berceni Road, 041915 Bucharest, Romania

**Keywords:** burn injury, quality of life, burn survivors, Burn Specific Health Scale-Brief (BSHS-B), psychological outcomes, functional recovery, depression, return to work, rehabilitation, post-burn sequelae

## Abstract

**Background:** Burn injuries are increasingly being recognized as chronic conditions with long-term physical, emotional, and social consequences. As survival after acute burn trauma improves, greater attention has shifted toward health-related quality of life (QoL) in survivors, particularly in regions where data remain limited. **Methods:** This study included burn survivors treated between January 2022 and December 2023 in the Department of Plastic Surgery and Reconstructive Microsurgery of the Emergency Clinical Hospital “Bagdasar-Arseni,” Bucharest, Romania. Patients who survived hospitalization and follow-up were invited to complete a Romanian-adapted version of the Burn Specific Health Scale-Brief (BSHS-B). Demographic and clinical data were collected from medical records, including burn type, total body surface area (TBSA), burn depth, burn localization, and access to rehabilitation services. Statistical analysis included descriptive methods, chi-square tests, *t*-tests, Kendall’s tau-b, Cramer’s V, Cronbach’s alpha, and exploratory factor analysis. **Results:** Thirty-eight patients were included. Most burns were thermal (94.74%), while burns involving <10% TBSA were most frequent (60.53%). Functional outcomes were generally favorable, with most patients reporting no difficulty in basic daily activities such as bathing, dressing, and writing. However, fine motor activities and return to previous work were more frequently affected. Emotional recovery appeared less complete, with persistent mild-to-moderate loneliness, sadness, and emotional distress reported by many participants. Women reported higher levels of loneliness (*p* = 0.015), while third-degree burns were associated with more frequent depressive symptoms (*p* = 0.008). Depressive symptoms were also significantly associated with functional limitations (such as getting dressed, *p* = 0.002) and work impairment (*p* < 0.001). The adapted functional and emotional subscales showed excellent internal consistency. **Conclusions:** Post-burn recovery extends beyond physical healing. Although most patients regained functional independence, emotional distress and occupational difficulties often persisted. These findings support the need for multidisciplinary long-term burn care integrating physical rehabilitation, psychological screening, and psychosocial support.

## 1. Introduction

Burns are defined as traumatic lesions of the skin and underlying tissues, caused by extreme heat, friction, contact with chemicals, electricity or radioactivity and radiation [1,2]. In 2021, global statistics reported a prevalence of 12.99 million severe burns and 235.34 million mild burns worldwide [3]. The region in which the present study is conducted—Eastern Europe—is projected to experience a 113% increase in the incidence of severe burns between 2030 and 2050, representing the highest rise among all global regions [3]. Acute mortality following burn injury is strongly influenced by patient-related factors such as age and sex, as well as by burn size and the presence of associated inhalation injury. In addition, severe burns may trigger a profound stress response characterized by a hypermetabolic state, increased susceptibility to infections and immunological impairment [4]. With advances in acute care, survival rates after burn injury have significantly improved over recent decades [5,6]. As a result, increasing attention has been directed toward the long-term sequelae of burn injury [7]. These factors collectively influence quality of life (QoL), defined by the World Health Organization (WHO) as an individual’s perception of their position in life in the context of the culture and value systems in which they live and in relation to their goals, expectations, standards and concerns [8].

Appropriate follow-up care for burn survivors should incorporate the assessment of these domains; accordingly, several scales and questionnaires have been developed, including the Brief Version of the Burn Specific Health Scale (BSHS-B), which provides a comprehensive evaluation encompassing domains such as Simple Functional Abilities, Work, Body Image, Interpersonal Relationships, Affect, Heat Sensitivity, and Treatment Regimens [9]. A recent analysis of 24 studies demonstrated considerable variability across countries and cultures in the impact of both major and minor burns on affect, body image, interpersonal relationships, and sexuality [10].

Consequently, burn injury should be regarded as a chronic condition, wherein long-term management aims to address the physical, psychological and social sequelae [11,12].

The objective of the present study is to further elucidate the medium- to long-term outcomes of burn injury, with a particular focus on health-related quality of life and the impact of burn sequelae on activities of daily living.

## 2. Methodology

This study was designed as an observational, retrospective, descriptive, and analytical study, involving the Department of Plastic Surgery and Reconstructive Microsurgery at the Emergency Clinical Hospital “Bagdasar-Arseni” in Bucharest, Romania. The study was conducted in accordance with the Declaration of Helsinki and approved by the Ethics Committee of the Emergency Clinical Hospital “Bagdasar-Arseni” (protocol number 20284/21 April 2026).

The inclusion criteria for the study was the diagnosis and treatment of burn injuries, mostly from thermal injuries, treated both conservatively and surgically, in the Burns Department, between January 2022 and December 2023, and age over 18 years old—as such, a time period of 3–4 years between accident and questionnaire was achieved and longer-term observations could be made. The exclusion criteria were patients who died during hospitalization or during the follow-up period. After following these criteria, an initial number of 147 patients were identified, who were contacted and proposed to provide data for the study. Of those, 127 patients accepted the invitation to participate and provided informed consent, but ultimately only 38 of them completed the questionnaire and were included in the final analysis (29.92% response rate). The questionnaire was self-administered, using Google Forms, and was applied in the 23 February 2026–1 March 2026 time period.

The data analysed in the present study was collected from medical records and directly from patients through a questionnaire. The patients’ medical records provided demographic data (gender, age) and data pertaining to the burn injury (localisation, total body surface area affected (TBSA), using the Lund and Browder chart [13], burn depth: second or third degree, access to physiotherapy and psychotherapy during hospital stay).

The questionnaire used was a version of the Burn Specific Health Scale–Brief (BSHS-B), adapted and translated into Romanian, and it was applied as a preliminary exploratory instrument. A full cross-cultural adaptation and validation process was not performed in the present study and remains to be conducted in future research. The final adapted instrument comprised 33 items and was administered to eligible participants. The questionnaire assesses multiple dimensions of post-burn quality of life, including hand function and simple abilities, affect, interpersonal relationships, sexuality, body image, heat sensitivity, treatment regimens, and return-to-work adaptation [9]. In the present study, the analysis focused specifically on the functional and emotional domains.

### Statistical Analysis

Statistical analyses were performed using IBM SPSS Statistics version 27 and Microsoft Excel 2019 MSO (Version 2508). Continuous variables were described as mean ± standard deviation (SD) and median, while categorical variables were summarized as frequencies and percentages. Group comparisons were conducted using the independent samples *t*-test, with normality assessed using the Kolmogorov–Smirnov and Shapiro–Wilk tests, as well as graphical analysis. Associations between categorical variables were evaluated using Fisher’s exact test (incorporating the Fisher–Freeman–Halton extension for tables larger than 2 × 2), and effect sizes were estimated using Kendall’s tau-b (τb) or Cramer’s V coefficients. Given the exploratory nature of this study, no formal correction for multiple comparisons was applied.

Before conducting the association analyses, several clinically driven a priori hypotheses were defined. First, greater burn severity, reflected by third-degree burns and the need for skin grafting, was expected to be associated with poorer functional outcomes and more frequent emotional symptoms. Second, functional limitations in daily activities, especially those involving self-care, fine motor skills, and return to previous work, were expected to be associated with higher levels of sadness or depressive symptoms. Third, impairment in professional reintegration was expected to correlate with greater emotional burden.

Associations involving demographic variables, individual emotional items, and the sexual difficulty item were considered exploratory. Given the limited sample size and the preliminary nature of the study, these analyses were used to identify clinically relevant patterns rather than to confirm definitive predictive relationships.

The items of the questionnaire were formulated using a 5-point Likert scale (ranging from “none” to “extreme”). In the present study, only items corresponding to the functional and emotional domains were analyzed. The internal consistency of these subscales was assessed using Cronbach’s alpha coefficient, and a preliminary exploratory factor analysis (principal component analysis with Varimax rotation based on a Pearson correlation matrix) was conducted. However, given the small sample size, this analysis was interpreted with caution and treated strictly as a descriptive indicator of item clustering, without constituting a formal psychometric validation.

Floor and ceiling effects were assessed at the subscale level by calculating the proportion of participants achieving the minimum and maximum possible scores for each analyzed subscale.

## 3. Results

### 3.1. Patient Characteristics

The study included 38 burn patients admitted between January 2022 and December 2023 to the Burns Unit of Bagdasar-Arseni Emergency Clinical Hospital in Bucharest, Romania.

Male patients predominated, accounting for 57.89% of cases (*n* = 22), while women represented 42.11% (*n* = 16). The age of participants at the time of questionnaire completion ranged from 22 to 81 years, with a mean age of 55.53 ± 15.36 years and a median age of 58.50 years. At the time of the burn injury, patients were aged between 19 and 77 years, with a mean age of 53.32 ± 15.16 years and a median age of 57 years. Men were significantly younger than women—see Table 1.

### 3.2. The Characteristics of Burn Injuries and Health Services Received

Most patients sustained thermal burns (94.74%, *n* = 36), while electrical burns (2.63%, *n* = 1) and combined thermal–chemical burns (2.63%, *n* = 1) were less frequent. Burns involving less than 10% of total body surface area (TBSA) were most common (60.53%, *n* = 23), followed by burns of 10–20% TBSA (26.32%, *n* = 10). Less frequent were burns involving 21–30% (5.26%, *n* = 2), 31–40% (5.26%, *n* = 2), and 51–60% TBSA (2.63%, *n* = 1). Regarding burn depth, 50% of cases were second-degree burns (*n* = 19), while the remaining 50% were third-degree burns (*n* = 19).

In terms of anatomical distribution, the most commonly affected regions were the hand (47.37%, *n* = 18), head (42.11%, *n* = 16), forearm (39.47%, *n* = 15), thigh (39.47%, *n* = 15), and leg (36.84%, *n* = 14)—see Table 2.

Following the burn injury, physiotherapy was recommended in 97.37% of cases (*n* = 37), while psychotherapy was recommended in 57.89% of cases (*n* = 22). All patients who received these recommendations adhered to and completed the prescribed therapies. Following questionnaire completion, 39.47% of patients (*n* = 15) reported having received specialized psychological support after the burn injury. Women were more likely to access such services compared to male patients (43.75% vs. 36.36%; *n* = 7 vs. *n* = 8). An increasing trend in the utilization of psychological support was observed with greater burn surface area involvement: 30.43% among patients with <10% TBSA burns (*n* = 7), 50% among those with 10–20% TBSA burns (*n* = 5), and 100% among patients with burns exceeding 30% TBSA (*n* = 3). Psychological support was also more frequently reported among patients with inhalation injuries (75%, *n* = 3) and among those with burns affecting the thorax (63.64%, *n* = 7), head (56.25%, *n* = 9), and forearm (53.33%, *n* = 8).

It should be noted that psychological support was self-reported by patients through the questionnaire and may reflect both interventions received during hospitalization and continued engagement with psychological services during the post-burn recovery period.

### 3.3. BSHS-B Application in a Romanian Cohort

As far as the authors are aware, the BSHS-B has not yet been formally validated in a Romanian population; therefore, only preliminary exploratory psychometric analyses were conducted in the present cohort. Given the limited sample size, these analyses should be interpreted with caution and cannot be considered a formal validation of the instrument. Both the functional subscale and the emotional subscale suggest excellent internal consistency (Cronbach’s alpha = 0.955 and 0.906, respectively) within this specific sample but cannot be generalized. A preliminary exploratory factor analysis using principal component analysis with Varimax rotation was performed—see Table 3. The Kaiser–Meyer–Olkin measure (KMO = 0.836) and Bartlett’s test (*p* < 0.001) formally met the statistical criteria for analysis, and a two-dimensional structure emerged, corresponding to functional and emotional domains, accounting for 82.15% of the total variance. This structure is consistent with the conceptual framework of the BSHS-B, but other domains of the BSHS-B were not analyzed; therefore, no conclusions can be drawn regarding the overall structure or validity of the full instrument.

#### 3.3.1. Functional Outcomes in the Post Burn Period

**Figure 1:**
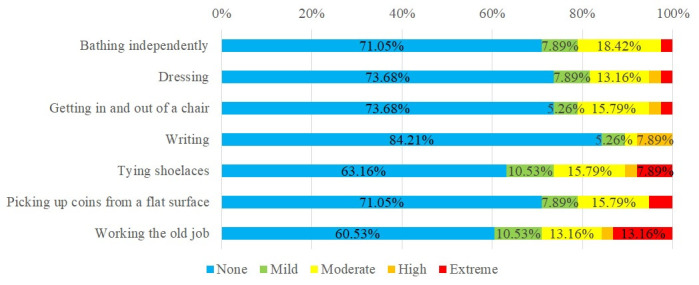
Difficulty in performing daily activities.

Overall, the results indicate a high level of functional autonomy in basic daily activities. Most patients reported no difficulties in performing self-care tasks: 71.05% (*n* = 27) reported no difficulty in bathing independently, 73.68% (*n* = 28) in dressing themselves, and 73.68% (*n* = 28) in getting in and out of a chair. Writing was not perceived as problematic by 84.21% of participants (*n* = 32). Moderate difficulties were reported by 13.16% (*n* = 5) of patients for dressing and 18.42% (*n* = 7) for bathing, while such difficulties were rarely reported for writing (2.63%, *n* = 1). Extreme difficulties in performing these activities were reported by 2.63% (*n* = 1) of patients, with no cases reported for writing (0%)—see Figure 1.

Activities requiring greater fine motor skills were slightly more affected. A total of 63.16% (*n* = 24) of patients reported no difficulty tying shoelaces, while 71.05% (*n* = 27) reported no difficulty picking up small objects (e.g., coins) from a flat surface. Moderate difficulties were reported by 15.79% (*n* = 6) of patients for each of these activities. However, extreme difficulties were more frequently observed in these domains, affecting 7.89% (*n* = 3) of patients for tying shoelaces and 5.26% (*n* = 2) for picking up small objects.

Among all evaluated activities, returning to the previous workplace had the lowest proportion of patients reporting no difficulties (60.53%, *n* = 23), this activity being the most frequently associated with extreme difficulties (13.16%, *n* = 5).

In this study, the need for skin grafting was interpreted as a marker of burn injury severity, as it generally reflected deeper burns requiring surgical wound closure and was associated with a more complex post-burn recovery profile. Skin grafting was required in 71.05% of patients (*n* = 27). The first skin grafting procedure was performed between the day of injury and 24 days after the burn, with a mean time to skin grafting of 5.67 ± 7.27 days and a median of 2 days. Among patients who underwent grafting, 33.33% (*n* = 9) received the procedure on the day of injury.

Although most associations did not reach statistical significance, patients who required skin grafting tended to report functional difficulties more frequently than those who did not. These difficulties were mostly moderate and involved activities such as washing (40.74%, *n* = 11), dressing (37.04%, *n* = 10), sitting down or standing up from a chair (37.04%, *n* = 10), tying shoelaces (48.15%, *n* = 13), and picking up coins from a flat surface (37.04%, *n* = 10). In contrast, patients who did not require grafting reported no difficulties with washing, dressing, standing up from a chair, or writing, while difficulties with tying shoelaces and picking up coins were reported by only one patient each (9.09%).

#### 3.3.2. Emotional Outcomes

**Figure 2:**
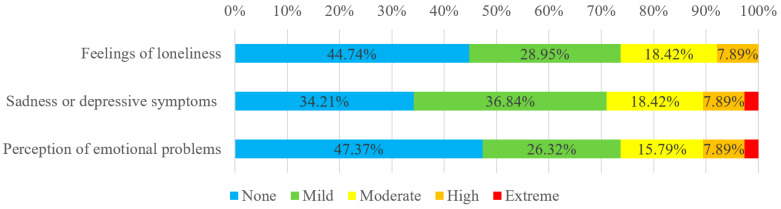
Evaluation of emotional outcomes.

Overall, the results indicate a low prevalence of severe emotional difficulties, with most patients reporting either no symptoms or only mild manifestations—see Figure 2. Feelings of loneliness were absent in 44.74% of participants (*n* = 17), while 28.95% (*n* = 11) reported mild, 18.42% (*n* = 7) moderate, and 7.89% (*n* = 3) high levels; no patients reported extreme loneliness (0%). Similarly, sadness or depressive symptoms were absent in 34.21% (*n* = 13) of patients and mild in 36.84% (*n* = 14), while 18.42% (*n* = 7) reported moderate symptoms, 7.89% (*n* = 3) severe symptoms, and 2.63% (*n* = 1) extreme symptoms.

Patients who underwent skin grafting showed more frequent emotional impairment. Among these patients, loneliness was reported as mild in 33.33% of cases (*n* = 9), moderate in 22.22% (*n* = 6), and severe in 11.11% (*n* = 3). By contrast, most patients who did not need skin grafting reported no loneliness (72.73%, *n* = 8), while mild and moderate loneliness were reported by 18.18% (*n* = 2) and 9.09% (*n* = 1), respectively.

Regarding the perception of emotional problems, nearly half of the patients (47.37%, *n* = 18) did not identify any such difficulties. Among those reporting emotional distress, most described it as mild (26.32%, *n* = 10) or moderate (15.79%, *n* = 6), while higher levels were less frequently reported (severe: 7.89%, *n* = 3; extreme: 2.63%, *n* = 1).

Notably, emotional difficulties appeared to be more prevalent than functional limitations, as reflected by the lower proportion of patients reporting complete absence of symptoms in the emotional domain compared to the functional domain.

Floor and ceiling effects were evaluated for the analyzed subscales. No floor effects were observed for either the functional or emotional subscales. However, ceiling effects were identified for both domains, affecting 50% (*n* = 19) of patients in the functional subscale and 31.58% (*n* = 12) in the emotional subscale.

#### 3.3.3. Correlation Between Gender and Loneliness

**Figure 3:**
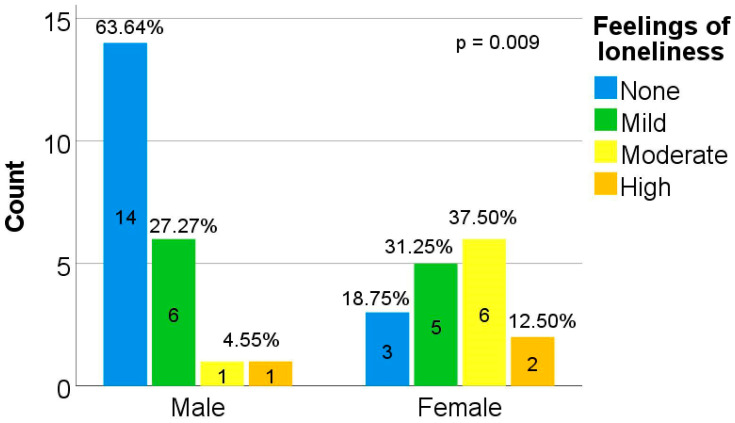
Loneliness according to the patient’s gender.

A statistically significant association was observed between patient gender and feelings of loneliness (Fisher’s exact test *p* = 0.009; Cramer’s V = 0.524, *p* = 0.015;)—see Figure 3. Women more frequently reported moderate (37.50% vs. 4.55%; *n* = 6 vs. *n* = 1) and higher levels of loneliness (12.50% vs. 4.55%; *n* = 2 vs. *n* = 1) than male patients, whereas the absence of loneliness was more commonly reported among male patients (63.64% vs. 18.75%; *n* = 14 vs. *n* = 3).

These findings may suggest greater emotional vulnerability among women in the post-burn period; however, they should be interpreted with caution given the relatively small cohort.

#### 3.3.4. Correlation Between Burn Depth and Frequency of Sadness and Depression Symptoms

**Figure 4:**
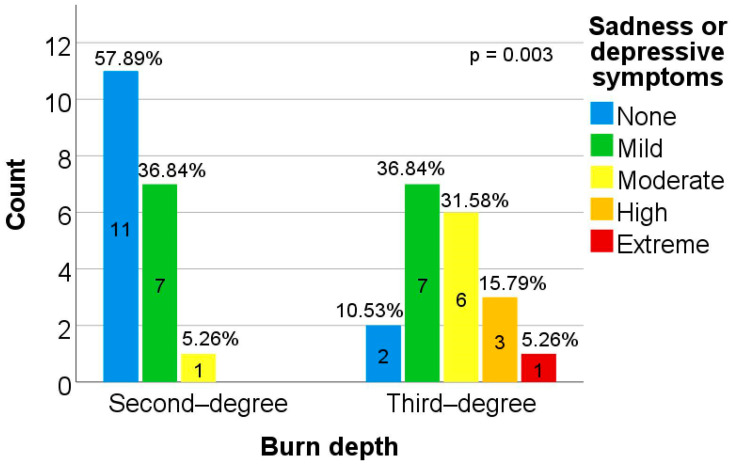
The level of sadness/depression according to burn depth.

A statistically significant association was observed between burn depth and the frequency of sadness/depressive symptoms (Fisher’s exact test *p* = 0.003, Cramer’s V = 0.603, *p* = 0.008;), with patients presenting deeper burns reporting more pronounced depressive symptomatology—see Figure 4.

Among patients with second-degree burns, the majority reported either absence (57.89%, *n* = 11) or low levels of sadness/depression (36.84%, *n* = 7), with no cases of severe symptoms identified. On the other hand, the answers of patients with third-degree burns shifted toward higher levels of emotional impairment: 31.58% reported moderate symptoms (*n* = 6), 15.79% severe symptoms (*n* = 3), and 5.26% extreme symptoms (*n* = 1), while only 10.53% reported absence of depressive symptoms (*n* = 2).

Sadness or depressive symptoms were significantly more frequent among patients who required skin grafting (Fisher’s exact test, *p* = 0.019). In this group, symptoms were reported as mild in 40.74% of cases (*n* = 11), moderate in 25.93% (*n* = 7), severe in 11.11% (*n* = 3), and extreme in 3.70% (*n* = 1). In the non-grafted group, absence of sadness or depressive symptoms predominated (72.73%, *n* = 8), and the remaining patients reported only mild symptoms (27.27%, *n* = 3). However, this association should be interpreted cautiously because of the small sample size.

#### 3.3.5. Correlation Between Functional Limitations of Daily Activities and Sadness/Depression

Depressive symptomatology was also significantly associated, with difficulties in performing basic functional activities, including dressing (Fisher’s exact test, *p* = 0.002), sitting down and standing up from a chair (Fisher’s exact test, *p* ≤ 0.001), writing (Fisher’s exact test, *p* = 0.012), tying shoelaces (Fisher’s exact test, *p* = 0.010), and picking up coins from a flat surface (Fisher’s exact test, *p* = 0.006). However, these associations should be interpreted as exploratory given the number of statistical tests performed and the small sample size.

Overall, patients who reported greater functional impairment were significantly more likely to exhibit moderate to high levels of sadness/depression, suggesting that reduced functional autonomy may contribute to emotional distress, as detailed below.

In the case of dressing, patients without difficulties predominantly reported low levels of sadness (none: 46.43%, *n* = 13; mild: 39.29%, *n* = 11), whereas those with moderate difficulties more frequently exhibited higher levels of emotional distress (high: 40%, *n* = 2; extreme: 20%, *n* = 1)—see Table 4. Patients with greater difficulties reported exclusively moderate levels of sadness/depression (100%, *n* = 1). A statistically significant correlation was identified between dressing difficulties and depressive symptomatology (Kendall’s τb = 0.516, *p* < 0.001).

In the case of bathing independently, the association between functional limitation and depressive symptoms did not reach statistical significance; however, a similar pattern was observed, with greater functional impairment tending to be accompanied by higher levels of sadness/depression.

For getting in and out of a chair, the absence of difficulties was associated with low levels of depressive symptomatology (none: 46.43%, *n* = 13; mild: 39.29%, *n* = 11)—see Table 4. By contrast, patients with moderate difficulties more frequently reported higher levels of emotional distress (mild: 50%, *n* = 3; high: 33.33%, *n* = 2; extreme: 16.67%, *n* = 1), while those with greater difficulties reported exclusively moderate levels of sadness/depression (100%, *n* = 1). A statistically significant correlation was identified between these functional limitations and depressive symptoms (Kendall’s τb = 0.506, *p* < 0.001).

Regarding writing ability, most patients without difficulties reported minimal levels of sadness (none: 40.63%, *n* = 13; mild: 37.50%, *n* = 12). By contrast, greater writing-related difficulties (category “high”) were associated exclusively with moderate levels of depressive symptoms (100%, *n* = 3). A statistically significant correlation was observed between writing impairment and depressive symptomatology (Kendall’s τb = 0.345, *p* = 0.012).

Regarding shoelace tying, patients without difficulties predominantly reported low levels of depressive symptoms (none: 45.83%, *n* = 11; mild: 41.67%, *n* = 10). By contrast, patients reporting severe difficulties exhibited higher levels of sadness/depression (100%, *n* = 1), while those with extreme difficulties presented moderate (66.67%, *n* = 2) or extreme (33.33%, *n* = 1) depressive symptoms. A statistically significant association was identified between impairment in shoelace tying and depressive symptomatology (Kendall’s τb = 0.437, *p* = 0.002).

A comparable pattern was observed for the manipulation of small objects (picking up coins from a flat surface). Patients without difficulties predominantly reported low levels of sadness (none: 44.44%, *n* = 12; mild: 44.44%, *n* = 12). However, as motor difficulties increased, a corresponding worsening of depressive symptomatology was observed. Patients with extreme functional impairment reported moderate (50%, *n* = 1) or high (50%, *n* = 1) levels of sadness/depression. A statistically significant association was identified between difficulty in manipulating small objects and depressive symptoms (Kendall’s τb = 0.502, *p* < 0.001).

#### 3.3.6. Correlation Between Sadness/Depression and Sexuality

**Figure 5:**
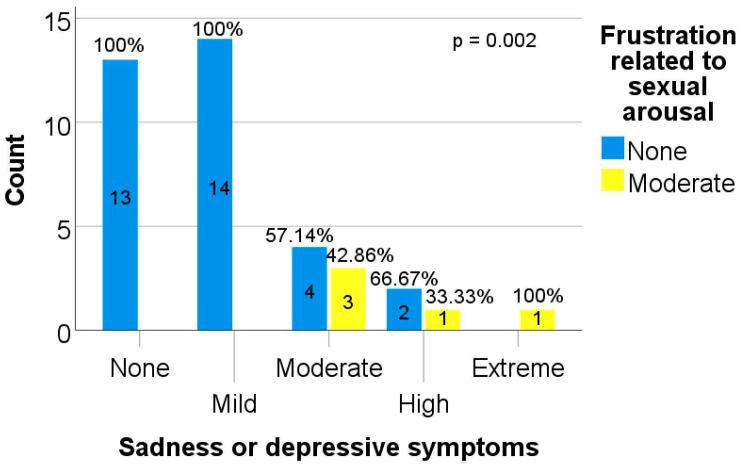
The link between depressive symptoms and sexuality.

A statistically significant association was observed between sexual difficulties (frustration related to the inability to achieve sexual arousal as before the burn injury) and depressive symptoms (Fisher’s exact test, *p* = 0.002)—see Figure 5. The relationship was direct (Kendall’s τb = 0.479, *p* = 0.006), suggesting a possible association between higher levels of sadness/depression and greater frequency of sexual frustration, but the cohort size was too small to draw firm conclusions.

Sexual frustration was reported more frequently among patients presenting higher levels of depressive symptoms (moderate: 42.86%, *n* = 3; severe: 33.33%, *n* = 1; extreme: 100%, *n* = 1). In contrast, sexual frustration was not reported among patients who did not experience depressive symptoms, suggesting that sexual difficulties were more common among patients with more pronounced depressive symptomatology.

#### 3.3.7. Correlation Between the Ability to Work and Sadness and Depression

A statistically significant association was observed between impairment in work capacity and the intensity of sadness/depressive symptoms (Fisher’s exact test, *p* < 0.001). The relationship was direct (Kendall’s τb = 0.511, *p* < 0.001), suggesting that a greater impact of burn injury on professional ability might be associated with higher levels of sadness or depression.

Patients reporting an extreme impact of burn injury on work capacity presented exclusively moderate levels of sadness (100%, *n* = 2). Among those reporting a severe impact, depressive symptoms were predominantly high (50%, *n* = 2) or moderate (25%, *n* = 1). In patients with moderate impairment, depressive symptoms were mainly moderate (66.67%, *n* = 2) or high (33.33%, *n* = 1).

For individuals reporting a minor impact on professional activity, the distribution was oriented toward lower levels of sadness/depression (mild: 50%, *n* = 3; none: 33.33%, *n* = 2), with one isolated case of extreme symptoms (16.67%, *n* = 1). In contrast, patients without impairment of work capacity predominantly reported minimal levels of sadness (none: 47.83%, *n* = 11; mild: 43.48%, *n* = 10).

## 4. Discussion

Greater burn severity, reflected by total body surface area (TBSA) and depth of injury, is generally linked to poorer QoL outcomes due to functional impairment, visible scarring and psychological distress, which in turn affect school or work reintegration, social life and sexual relationships. However, injury severity alone does not fully explain variability in long-term adjustment [14]. Length of hospital stay (LOS) has long been seen as a particularly relevant predictor of QoL. While strongly correlated with TBSA, LOS appears to have an independent and stronger association with QoL, influencing interpersonal relationships, work status and time to functional recovery [14]. Furthermore, the patient’s support system strongly shapes QoL outcomes [14].

The present study provides a comprehensive evaluation of post-burn quality of life in a Romanian cohort and represents, to the authors’ knowledge, the first application of the Burn Specific Health Scale–Brief (BSHS-B) in this population. Similar studies have shown that evaluation through the BSHS-B scale is a reliable instrument for assessing long term quality of life in burn patients across different populations [15,16,17,18,19,20]. The internal consistency suggests acceptable reliability within this sample; however, these findings are only preliminary and do not establish the validity of the instrument in the Romanian population. Furthermore, significant ceiling effects were observed, particularly for the functional subscale, suggesting that the instrument may possess limited sensitivity in discriminating among patients with higher recovery levels. This finding aligns with the high proportion of patients reporting minimal or no functional limitations and may reflect a clustering of scores at the upper end of the scale. Given the relatively long follow-up period and generally favorable functional outcomes, these ceiling effects might also reflect the inherent characteristics of the study population rather than mere instrumental limitations.

The main finding of this study is the discrepancy between functional and emotional recovery following burn injury. While most patients achieved a high level of independence in activities of daily living, emotional recovery appeared less complete. Although severe psychological symptoms were uncommon, a considerable proportion of patients reported persistent mild-to-moderate emotional distress. Similar findings have been reported in previous studies, where burn survivors continued to experience emotional distress despite good physical recovery [21,22]. This imbalance suggests that physical rehabilitation outcomes may overestimate overall recovery. By contrast, psychological adaptation appears to be slower and less visible, emphasizing the need for systematic evaluation of emotional health even in patients with good functional outcomes.

Although basic autonomy was largely preserved, some residual functional limitations remained, particularly in activities requiring fine motor control, such as writing, tying shoelaces, or handling small objects. These tasks, while seemingly minor, may reflect subtle deficits related to scar stiffness, reduced dexterity or even neuropathic involvement. Similar results have been reported in studies on long-term burn outcomes, where survivors continued to experience difficulties in performing fine motor activities despite functional independence [23,24,25]. In this context, it becomes important to distinguish between achieving independence and achieving full functional recovery.

While skin grafting itself should not be interpreted as the direct cause of poorer functional or emotional outcomes, the need for skin grafting may be regarded as an indicator of greater burn severity. Patients requiring skin grafting likely had deeper lesions and a more complex recovery process, which may partly explain the higher frequency of functional limitations and emotional symptoms observed in this subgroup.

Among all functional domains, return to work stood out as the most affected. More than half of the patients reported some degree of difficulty in resuming their previous professional activities. This finding is not surprising, as returning to work involves more than physical ability; it also requires cognitive adaptation, emotional stability, and social reintegration. Emotional distress, pain and social isolation have been shown to significantly reduce work reintegration after burn injury [26]. The strong association identified between work impairment and depressive symptoms further supports the idea that occupational reintegration plays a central role in recovery. In many cases, inability to return to work may contribute to a sense of loss of identity and reduced self-worth, which can sustain emotional distress [27].

Emotional outcomes in this study were characterized by persistent low-to-moderate levels of distress and less by severe pathology. The proportion of patients reporting complete absence of emotional symptoms was smaller than the proportion of patients reporting no functional limitations. This difference is clinically relevant, as it suggests that emotional burden may remain underrecognized, particularly if attention is focused only on severe psychiatric manifestations. Even mild or moderate levels of emotional distress can significantly affect quality of life, self-perception and social reintegration after burn injuries as previous studies also have shown [28,29].

Gender differences were also observed, with women reporting higher levels of loneliness. While this finding aligns with some existing observations in the literature [30], it should be interpreted cautiously given the limited sample size. Possible explanations include differences in body image perception, social roles or patterns of emotional expression, but these remain speculative in the context of the present data.

Burn severity also appeared to influence emotional outcomes. Patients with third-degree burns reported higher levels of depressive symptoms compared to those with more superficial injuries. This may be related to the more pronounced physical consequences of deeper burns, including scarring and functional impairment, as well as the longer and more complex treatment process. These factors likely interact with psychological adaptation, particularly through their impact on body image and self-perception and social integration [28,29,31].

An important aspect of the present findings is the consistent association between functional impairment and depressive symptoms. Patients who experienced greater difficulty in performing daily activities were more likely to report higher levels of sadness or depression. This relationship is likely bidirectional. On the one hand, reduced autonomy may negatively affect self-efficacy and increase psychological vulnerability [27]. On the other hand, depressive symptoms may influence how patients perceive and report their functional abilities [32,33].

Notably, even relatively minor impairments, particularly those involving fine motor skills, were associated with increased emotional distress. Activities such as writing or tying shoelaces are performed frequently and require precision, which may make any limitation more noticeable and, potentially, more frustrating [23,34]. As such, these seemingly small deficits may have a disproportionate psychological impact [25].

A similar relationship was observed between depressive symptoms and sexual difficulties. Although the reported prevalence of sexual impairment was relatively low, this finding should be interpreted with caution, as this domain is likely underreported. Sexual function is closely linked to body image, self-esteem and interpersonal relationships, all of which may be affected following burn injury [35,36,37,38].

Overall, the results point to a mismatch between physical and psychosocial recovery. Patients may regain functional independence while continuing to experience emotional distress, social difficulties or challenges in returning to work. Recovery after burn injury should therefore be understood as a multidimensional process, rather than a purely physical one [31,34,39,40].

From a clinical perspective, these findings support the need for a more integrated approach to burn care. In addition to physical rehabilitation, attention should be given to psychological screening and support, particularly in patients with more severe injuries, functional limitations or difficulties in professional reintegration. Similar recommendations have been emphasized in previous studies [41,42,43]. Structured occupational rehabilitation and long-term psychosocial support may play an important role in improving overall outcomes.

Importantly, the questionnaire was applied at an average 3.5 years after hospitalisation for burn injury, revealing a medium-term snapshot of the functional and psychological sequelae of burns. Similarly, other studies reveal a lasting impact of the accident: a study which involved over 80% of participants at least one year away from hospitalisation showed a lasting impact on the scale of depression/anxiety and pain/discomfort [44]. Even ten years after injury, the BSHS scale revealed moderate severity of injury, with lower scores in heat sensitivity, affect, body image, and work [45].

Several limitations should be acknowledged. The relatively small sample size and low response rate limits the generalizability of the findings and reduces the robustness of subgroup analyses. This is particularly relevant for certain observations, such as those related to specific anatomical regions (e.g., shoulder girdle involvement), where the number of cases was very limited. In addition, the study combines retrospective data from medical records with self-reported questionnaire data, which introduces the possibility of reporting bias, especially in sensitive areas such as emotional health and sexuality. The cross-sectional design also prevents any inference of causality, and the lack of pre-burn baseline data limits the ability to quantify changes over time.

Although several of the observed associations were statistically significant and clinically plausible, they should be interpreted within the framework of the predefined hypotheses and the exploratory nature of the study. The small sample size, the use of multiple item-level comparisons, and the absence of correction for multiple testing limit the strength of inference. Therefore, these findings should be regarded as hypothesis-generating and require confirmation in larger prospective cohorts.

Finally, although the adapted BSHS-B showed excellent internal consistency, it has not been previously validated in the Romanian population. Despite these limitations, the consistency of the associations observed across multiple domains supports the relevance of the findings. However, one advantage of the present work is the longer follow-up—a 2018 systematic review of health-related QoL after burns revealed that only 34% of studies used longitudinal designs with multiple assessment points, which varied widely. The most common assessment times were during hospital admission and at 3, 6, 12 and 24 months post-injury. Although evidence suggests continued improvement in HRQoL after two years, high attrition rates complicate long-term follow-up [28], while our cohort presents the status of patients at over 24 months after hospitalisation, revealing the longer-term impact of burns on QoL.

## 5. Conclusions

Post-burn recovery should not be assessed solely in terms of functional independence. Quality of life after burn injuries is influenced both by functional limitations and by emotional impairment, the latter being more common. Psychological and social dimensions appear to recover more slowly and may persist even in patients with good physical outcomes, while functional limitations, particularly those related to professional reintegration, have been significantly associated with depressive symptoms. A more comprehensive, multidisciplinary approach is therefore essential in order to address the full spectrum of post-burn sequelae.

## Figures and Tables

**Table 1:** Demographic characteristics of the cohort.

Parameter	Value	*p*-Value
Number of patients (*n*, %)	Total: 38 Male: 22 (57.89%) Female: 16 (42.11%)	–
Age at questionnaire (years old, mean ± SD, median, range)	Total: 55.53 ± 15.36; 58.5 (22–81) Male: 49.50 ± 15.10; 46.0 (22–77) Female: 63.81 ± 11.66; 65.5 (39–80)	*p* = 0.003
Age at burn injury (years old, mean ± SD, median, range)	Total: 53.32 ± 15.16; 57.0 (19–77) Male: 47.86 ± 15.51; 43.0 (19–73) Female: 60.81 ± 11.26; 63.0 (36–77)	*p* = 0.007
Duration since burn injury (years, mean ± SD, median, range)	3.53 ± 0.50; 4.0 (3–4)	–

**Table 2:** Characteristics of burn injuries.

Parameter	Value
Type of burn (*n*, %)	Thermal: 36 (94.74%) Electrical: 1 (2.63%) Thermal + Chemical: 1 (2.63%)
Total burn surface area (TBSA) (*n*, %)	<10%—23 (60.53%) 10–20%—10 (26.32%) 21–30%—2 (5.26%) 31–40%—2 (5.26%) 51–60%—1 (2.63%)
Burn depth (*n*, %)	Second degree—19 (50%) Third degree—19 (50%)
Area of burn injuries (*n*, %)	Hand—18 (47.37%) Head—16 (42.11%) Forearm—15 (39.47%) Thigh—15 (39.47%) Leg—14 (36.84%) Foot—12 (31.58%) Thorax—11 (28.95%) Arm—11 (28.95%) Pelvis—9 (23.68%) Knee—9 (23.68%) Abdomen—7 (18.42%) Shoulder girdle—4 (10.53%) Fingers—4 (10.53%) Inhalation injury—4 (10.53%) Neck—3 (7.89%) Combined inhalation injury with smoke/toxic gas—1 (2.63%) Toes—1 (2.63%)

**Table 3:** Table Factor loadings after Varimax rotation for functional and emotional subscales.

Item	Functional Factor	Emotional Factor
Bathing independently	0.882	0.3
Dressing	0.875	0.318
Getting in and out of a chair	0.823	0.295
Writing	0.856	0.083
Tying shoelaces	0.861	0.338
Picking up coins from a flat surface	0.87	0.293
Working the old job	0.805	0.329
Feelings of loneliness	0.234	0.847
Sadness or depressive symptoms	0.33	0.885
Perception of emotional problems	0.242	0.894

**Table 4:** The link between functional limitations and sadness/depression.

		I Often Feel Sad or Depressed					Total
		Extreme	High	Moderate	Mild	None	
How difficult is it for you to bathe independently?	Extreme	0	0	1	0	0	1
	Moderate	1	2	2	2	0	7
	Mild	0	0	1	2	0	3
	None	0	1	3	10	13	27
How difficult is it for you to dress by yourself?	Extreme	0	0	1	0	0	1
	High	0	0	1	0	0	1
	Moderate	1	2	0	2	0	5
	Mild	0	0	2	1	0	3
	None	0	1	3	11	13	28
How difficult is it for you to get in and out of a chair?	Extreme	0	0	1	0	0	1
	High	0	0	1	0	0	1
	Moderate	1	2	0	3	0	6
	Mild	0	0	2	0	0	2
	None	0	1	3	11	13	28
How difficult is it for you to write?	High	0	0	3	0	0	3
	Moderate	0	1	0	0	0	1
	Mild	0	0	0	2	0	2
	None	1	2	4	12	13	32
How difficult is it for you to tie shoelaces?	Extreme	1	0	2	0	0	3
	High	0	1	0	0	0	1
	Moderate	0	1	1	2	2	6
	Mild	0	0	2	2	0	4
	None	0	1	2	10	11	24
How difficult is it for you to pick up coins from a flat surface?	Extreme	0	1	1	0	0	2
	Moderate	1	1	2	1	1	6
	Mild	0	0	2	1	0	3
	None	0	1	2	12	12	27
How difficult is it for you to work in your old job, performing your old duties?	Extreme	0	1	3	1	0	5
	High	1	0	0	0	0	1
	Moderate	0	2	0	2	1	5
	Mild	0	0	2	0	2	4
	None	0	0	2	11	10	23

## Data Availability

The datasets generated and/or analyzed during the current study are available from the corresponding author on reasonable request.

## Abbreviations

BSHS-B	Brief Version of the Burn Specific Health Scale
LOS	Length of hospital stay
QoL	quality of life
SD	standard deviation
TBSA	Total body surface area affected
WHO	World Health Organisation
